# Satellite cells in human skeletal muscle plasticity

**DOI:** 10.3389/fphys.2015.00283

**Published:** 2015-10-21

**Authors:** Tim Snijders, Joshua P. Nederveen, Bryon R. McKay, Sophie Joanisse, Lex B. Verdijk, Luc J. C. van Loon, Gianni Parise

**Affiliations:** ^1^Department of Kinesiology and Medical Physics and Applied Radiation Sciences, McMaster UniversityHamilton, ON, Canada; ^2^Department of Human Biology, NUTRIM School of Nutrition and Translational Research in Metabolism, Maastricht UniversityMaastricht, Netherlands

**Keywords:** muscle satellite cells, aging, exercise, myostatin, interleukin-6, IGF-1, Pax7, muscle fiber hypertrophy

## Abstract

Skeletal muscle satellite cells are considered to play a crucial role in muscle fiber maintenance, repair and remodeling. Our knowledge of the role of satellite cells in muscle fiber adaptation has traditionally relied on *in vitro* cell and *in vivo* animal models. Over the past decade, a genuine effort has been made to translate these results to humans under physiological conditions. Findings from *in vivo* human studies suggest that satellite cells play a key role in skeletal muscle fiber repair/remodeling in response to exercise. Mounting evidence indicates that aging has a profound impact on the regulation of satellite cells in human skeletal muscle. Yet, the precise role of satellite cells in the development of muscle fiber atrophy with age remains unresolved. This review seeks to integrate recent results from *in vivo* human studies on satellite cell function in muscle fiber repair/remodeling in the wider context of satellite cell biology whose literature is largely based on animal and cell models.

## Introduction

Adaptation is broadly defined as a change in behavior, physiology or structure in response to a changing environment. Adaptation in the context of skeletal muscle is often discussed as a change in structure and function in response to an exercise stimulus. The nature of adaptation in skeletal muscle in response to exercise is wholly dependent on the nature of the stimulus. For instance, resistance exercise training is generally characterized by increases in muscle mass and muscle fiber cross sectional area designed to resist the stress of lifting heavy loads. In contrast, endurance exercise training is generally characterized by metabolic adaptations designed to enhance fuel selection and ultimately resist fatigue. Although the concept of adaptation is rather simple, the processes that govern adaptation are significantly complex and involve interactions between multiple organs, tissues, cells, sub cellular organelles, and a plethora of coordinated signaling events. Skeletal muscle stem cells, also known as satellite cells, are now thought to play a significant role in the adaptive process.

Since their discovery by Mauro (1961) satellite cells have been identified as the main source of new myonuclei in postnatal skeletal muscle tissue (Reznik, 1969; Moss and Leblond, 1970). The term “satellite cell” was coined owing to the anatomical location of these cells between the sarcolemma and basal lamina of their associated muscle fiber. In adult muscle, satellite cells typically reside in a quiescent state; however, upon stimulation they activate, proliferate and/or differentiate. Myoblasts, the progeny of satellite cells, can either fuse with each other forming new myofibers, fuse to an existing muscle fiber donating their nucleus to the fiber, or return to the quiescent state, replenishing the resident pool of satellite cells through self-renewal (Schmalbruch, 1976). The progression of the satellite cell through the myogenic program is thought to be orchestrated by the up- or down-regulation of the paired box transcription factor 7 (Pax7) and the myogenic regulatory factors (MRFs) (Seale et al., 2000). From both *in vitro* cell and *in vivo* animal work it has been well-established that the up-regulation of Myf5 marks the earliest phase of myogenic commitment followed by the concomitant expression of MyoD, which marks the majority of newly activated satellite cells (Grounds et al., 1992; Smith et al., 1994; Cornelison and Wold, 1997; Cooper et al., 1999; Cornelison et al., 2000). Following proliferation, terminal differentiation of the satellite cell is believed to be initiated by the up-regulation of MRF4 and myogenin (Grounds et al., 1992; Smith et al., 1994; Yablonka-Reuveni and Rivera, 1994; Cornelison and Wold, 1997; Cornelison et al., 2000), and down-regulation of Pax7 (Olguin and Olwin, 2004; Olguin et al., 2007). However, when Pax7 expression remains elevated following proliferation, satellite cells exit terminal differentiation, and return to the quiescent state, thereby promoting self-renewal and maintenance of the basal satellite cell pool (Olguin and Olwin, 2004; Olguin et al., 2007).

Skeletal muscle satellite cells have been investigated using numerous *in vitro* and *in vivo* animal models to assess their role in muscle fiber maintenance, regeneration, and/or growth. However, in recent years, substantial effort has been made to translate these results from cell and animal work to the human model. In human skeletal muscle, the function and regulation of satellite cells is primarily investigated by using acute damaging or non-damaging exercise as a form of stress to mobilize the satellite cell population. These studies provide crucial information on the underlying mechanisms of satellite cell function under physiological conditions in humans. In this review we will discuss the identification of satellite cells in human skeletal muscle and provide a “signature” for the resting satellite cell pool. In addition, we will discuss the regulation of satellite cells during muscle fiber repair and remodeling in human skeletal muscle. We will describe factors currently considered to play a role in the process of satellite cell activation, proliferation, and/or differentiation in both animals and humans. Finally, we will discuss the impact of aging on satellite cell number and function and suggest future study directions.

## Satellite cell identification in human skeletal muscle

Due to its anatomical location, identification of satellite cells originally relied on electron microscopy, and all cells that were located beneath the basal lamina, and above the sarcolemma of a myofiber were considered satellite cells (Mauro, 1961). However, relatively recent advances in immuno-staining against various molecular markers has made the identification of satellite cells possible using light and/or immunofluorescent microscopy. In human skeletal muscle, the first antibody that was used to identity satellite cells by light microscopy was a glycoprotein called Leu-19 (Schubert et al., 1989). In this study satellite cells were identified by a “spike-like projection” of the Leu-19 antigen, which was not found around myonuclei and, secondly, they were localized beneath the basal lamina (Schubert et al., 1989). Subsequent studies showed that the Leu-19, neural cell adhesion molecule (NCAM) and, CD56 antigens have identical immunohistological labeling and staining patterns (Lanier et al., 1989; Illa et al., 1992; Mechtersheimer et al., 1992). The NCAM/CD56 antigen has been most frequently used to identify satellite cells in human skeletal muscle cryosections (Kadi et al., 1999; Kadi and Thornell, 2000; Renault et al., 2002; Charifi et al., 2003; Crameri et al., 2004; Kadi et al., 2004a,b,c; Dreyer et al., 2006; Kadi et al., 2006; Olsen et al., 2006; Petrella et al., 2006; Crameri et al., 2007; Mackey et al., 2007a,b; Verdijk et al., 2007; O'Reilly et al., 2008; Petrella et al., 2008; Verney et al., 2008; Lindstrom and Thornell, 2009; Mackey et al., 2009; Mikkelsen et al., 2009; Verdijk et al., 2010; Mackey et al., 2011a; Snijders et al., 2011, 2012; Theriault et al., 2012; Verdijk et al., 2012; Cermak et al., 2013; Leenders et al., 2013; Wernbom et al., 2013; Dirks et al., 2014a,b; Mackey et al., 2014; Snijders et al., 2014a; Theriault et al., 2014; Verdijk et al., 2014). Cells located at the periphery of myofibers, showing NCAM/CD56 staining around a nucleus, are considered satellite cells. Although the use of NCAM/CD56 is considered to be a reliable molecular marker to identify satellite cells in human skeletal muscle, this membrane bound protein is also expressed in myoblasts, myotubes, and muscle fibers during development and/or regeneration (Illa et al., 1992). Furthermore, NCAM/CD56 has been documented to stain positive along unmyelinated intramuscular nerves, at the surface of motor nerve terminals and Schwann cells (Cashman et al., 1987; Mechtersheimer et al., 1992). Alternative markers used to identify satellite cells in human skeletal muscle are the cell adhesion protein M-cadherin (M-Cad) (Reimann et al., 2004; Sajko et al., 2004), c-Met (Lindstrom et al., 2010; McKay et al., 2010), and Pax7 (Verdijk et al., 2007; Lindstrom and Thornell, 2009; Mackey et al., 2009; Mikkelsen et al., 2009; McKay et al., 2010). In animal muscle, M-Cad is a well-established marker of satellite cells (Irintchev et al., 1994). Irintchev et al. (1994) showed that virtually all M-Cad positive satellite cells co-localized with NCAM in mouse muscle. Interestingly, whereas the most intense staining of NCAM/CD56 is typically observed on the side of the satellite cell facing the muscle, M-Cad is also observed to stain between the satellite cell and parent muscle fiber. To date, only a few studies have used M-Cad as a molecular marker to successfully detect satellite cells in human skeletal muscle (Reimann et al., 2004; Sajko et al., 2004). To what extent M-Cad and NCAM/CD56 co-localize in human skeletal muscle remains unknown.

As a receptor for hepatocyte growth factor (HGF), c-Met has been shown to be present in both quiescent and activated satellite cells (Cornelison and Wold, 1997). Previous studies have used c-Met to identify human myogenic cells *in vitro* and *in vivo* as a total satellite cell marker (De Luna et al., 2004; Antunes-Foschini et al., 2006). So far, two studies have investigated the viability of the c-Met antigen to identify satellite cells in human skeletal muscle. In resting muscle biopsy samples from healthy young individuals, our laboratory has shown that the number of c-Met^+^/Pax7^+^ cells is significantly lower compared with NCAM^+^ or PAX7^+^ or NCAM^+^/PAX7^+^ cells (McKay et al., 2010). This would suggest that either c-Met expression is very low in quiescent human satellite cells or they represent a sub-population of the quiescent satellite cell pool. In addition, c-Met has also been shown to mark small blood vessels, myofiber membranes, and mononuclear interstitial cells in human skeletal muscle (Lindstrom et al., 2010). Both these *in vivo* human studies (Lindstrom et al., 2010; McKay et al., 2010) concluded that c-Met should not be considered as a reliable marker for satellite cell enumeration in human muscle cryosections.

Unlike NCAM, M-Cad, and c-Met, Pax7 is known to be expressed in the nuclei of myogenic progenitor cells during development and is thought to be exclusively expressed in satellite cells of mature muscle (Seale et al., 2000; Oustanina et al., 2004; Kuang and Rudnicki, 2008). In animal studies, the Pax7 antigen is the most widely used marker to identify satellite cells (Olguin and Olwin, 2004; Kuang et al., 2006; Relaix et al., 2006; Zammit et al., 2006; Day et al., 2007; Ishido et al., 2009; Lepper et al., 2011; McCarthy et al., 2011; Murphy et al., 2011; Sambasivan et al., 2011; Jackson et al., 2012) and has been proposed to be the most reliable marker for satellite cells in mouse muscle (Kuang and Rudnicki, 2008; Zammit, 2008). In the past decade, an increasing number of human studies have utilized the Pax7 antibody for the identification of skeletal muscle satellite cells *in vivo* (McLoon and Wirtschafter, 2003; Reimann et al., 2004; Verdijk et al., 2007; McKay et al., 2008; Mackey et al., 2009; McKay et al., 2009; Kottlors and Kirschner, 2010; McKay et al., 2010; Mackey et al., 2011a; Toth et al., 2011; McKay et al., 2012; Menon et al., 2012; Nielsen et al., 2012; Theriault et al., 2012; Walker et al., 2012; Bankole et al., 2013; Cermak et al., 2013; Delhaas et al., 2013; Joanisse et al., 2013; McKay et al., 2013; Suetta et al., 2013; Bellamy et al., 2014; Farup et al., 2014a,b; Fry et al., 2014a; Grubb et al., 2014; Kern et al., 2014; Mackey et al., 2014; Snijders et al., 2014b,c; Theriault et al., 2014). Reimann et al. (2004) compared M-Cad and Pax7 in biopsies taken from healthy and pathological human muscle. As M-Cad staining identified more cells in the satellite cell position than Pax7, the authors of this study advised against the use of Pax7 as an individual marker of satellite cells in human skeletal muscle. However, to date a widely accepted M-cad antibody is not available and more research into the characteristics of M-cad on human muscle satellite cells may be warranted. A number of other studies have investigated the use of NCAM/CD56 compared with Pax7 for satellite cell enumeration in humans (Verdijk et al., 2007; Lindstrom and Thornell, 2009; Mackey et al., 2009; Mikkelsen et al., 2009; McKay et al., 2010). Whereas, some studies show higher percentage (~5%) of cells in the satellite cell position expressing NCAM compared with Pax7 (Lindstrom and Thornell, 2009; Mackey et al., 2009; Mikkelsen et al., 2009), we have reported a slightly higher number of Pax7^+^ compared to NCAM^+^/CD56^+^ satellite cells in human muscle cross-sections (Verdijk et al., 2007; McKay et al., 2010). Although the reported differences between the number of Pax7^+^ and NCAM^+^/CD56^+^ satellite cells are small, some of this discrepancy may be attributed to the fact that NCAM/CD56 has also been shown to mark satellite cells committed to differentiation (Capkovic et al., 2008). In addition, *in vitro* studies indicate that NCAM is mainly involved in early myoblast differentiation of cell-cell fusion leading to enhanced myotube formation (Suzuki et al., 2003). The observation that NCAM/CD56 is expressed to a higher degree in terminal differentiating satellite cells may explain why studies have reported NCAM^+^/PAX7^−^ cells in human muscle biopsy samples (Lindstrom and Thornell, 2009; Mackey et al., 2009; Mikkelsen et al., 2009). As the majority of satellite cells are believed to progress toward terminal differentiation they may down-regulate Pax7 and up-regulate NCAM to allow for differentiation to occur (Capkovic et al., 2008; Kuang and Rudnicki, 2008). Hence, depending on the muscle biopsy sampling time point, the satellite cell pool may differentially express NCAM/CD56, and Pax7.

The use of any single molecular maker to identify satellite cells in human muscle cryosections for enumeration may inherently underestimate the total satellite cell pool size. Nonetheless, the use of laminin to identify the satellite cell niche appears to be critical, especially when using NCAM/CD56 as a satellite cell marker (Lindstrom and Thornell, 2009; Lindstrom et al., 2010). Multiple labeling (Pax7 and NCAM) will most likely provide the optimal identification of the satellite cell pool size at rest and in response to stimulation. However, the use of multiple nuclear markers in immunohistochemistry can become very difficult, especially in combination with additional cellular markers for satellite cell activation status.

Irrespective of the satellite cell marker used, it is important to note that satellite cell content cannot be reliably estimated on a small number of muscle fibers. Mackey et al. (2009) have shown that at least 50 type I and 75 type II muscle fibers are required to make a reliable estimation of fiber type specific satellite cell content in healthy young men. Whether the same number of muscle fibers is enough to make a reliable estimation of satellite cell content in other populations remains to be established, especially as satellite cell content has been suggested to decrease with advancing age (Verney et al., 2008; Carlson et al., 2009; Verdijk et al., 2009, 2010, 2012, 2014; McKay et al., 2012, 2013; Leenders et al., 2013; Suetta et al., 2013; Mackey et al., 2014; Snijders et al., 2014c). Counting all satellite cells in a muscle section (thereby increasing the number of fibers included in the analyses) is preferred as this will prevent subjective selection of the area being assessed. Furthermore, recent studies have emphasized the need for muscle fiber type specific analyses of satellite cell number. Resting satellite cell content has been shown to vary between muscle fiber types in both healthy and diseased populations (Verdijk et al., 2007, 2009, 2012; Verney et al., 2008; Snijders et al., 2011, 2014c; McKay et al., 2012; Suetta et al., 2012; Bankole et al., 2013). Furthermore, satellite cells have been shown to respond differently during post-exercise recovery depending on the muscle fiber type in which they are located (McKay et al., 2012; Cermak et al., 2013; Joanisse et al., 2013; Fry et al., 2014a; Snijders et al., 2014c).

When evaluating the satellite cell response to a stimulus, cell cycle analysis is of particular importance. Different molecular markers have been used to assess the changes in satellite cell activation status in response to various stimuli. The Ki-67 antigen is regarded as a marker of proliferating cells and is expressed during the mid-G1phase, with increased expression during the S and G2 phase and peaking in the M phase of the cell cycle (Gerdes et al., 1984). Whereas, some do (Mackey et al., 2009) others do not (Snijders et al., 2012; Cermak et al., 2013; Mackey et al., 2014) show a change in the number of Ki-67+ satellite cells after an anabolic stimulus in healthy young men. This discrepancy can most likely be explained by the relative short half-life of Ki-67, which makes the timing of muscle biopsy sampling critical to detect a change. Similarly, the proliferating cell nuclear antigen (PCNA) has been used to distinguish “active” from “non-active” satellite cells in human skeletal muscle (McKay et al., 2009; Cermak et al., 2013). However, PCNA is expressed in the nuclei of cells only in the DNA synthesis phase (S-phase) and late G1 phase, not the G2 and/or M phase of the cell cycle, and therefore may underestimate the number of active cells in a muscle cross-section. As both Ki-67 and PCNA are apparent in the nucleus during multiple or all stages of the cell cycle it does not discriminate between cells in different phases of the cell cycle. Flow cytometry is routinely used to analyse cells based on multiple antigen labeling *in vitro* and is used extensively in stem cell research to purify rare cell populations from a larger cellular milieu. Flow cytometry utilizes many DNA-specific markers such as Propidium Iodide to allow for a more precise measurement of cell cycle kinetics based on the DNA content of each cell. Hence, this allows for the description of the proliferation characteristics of satellite cells as they were *in vivo*, allowing for the determination of the number of cells in different phases of the cell cycle. Using flow cytometry, our laboratory has recently demonstrated that the number of satellite cells in G_0_/G_1_ of the cell cycle increases significantly during 24 and 48 h of recovery from a single bout of resistance and eccentric exercise, respectively, suggesting that within 24 h a proportion of satellite cells completed at least one round of cell division (McKay et al., 2010, 2012). This finding was supported by a significant increase in the number of Pax7^+^ cells per milligram tissue at 24 (McKay et al., 2010) and 48 h (McKay et al., 2012) after a single bout of eccentric or resistance type exercise, respectively. Furthermore, we have shown a significant increase in the number of satellite cells in the S-phase and G_2_/M phase of the cell cycle during the first 48 h of post-exercise recovery (McKay et al., 2010, 2012). Hence, flow cytometry is an objective, reproducible and precise measurement to investigate satellite cell cell-cycle kinetics in human skeletal muscle in response to exercise (McKay et al., 2010, 2012). Flow cytometry may also offer an objective way of sub-classifying the satellite cell pool based on different cell surface markers such as NCAM and M-cad.

**Figure 1:**
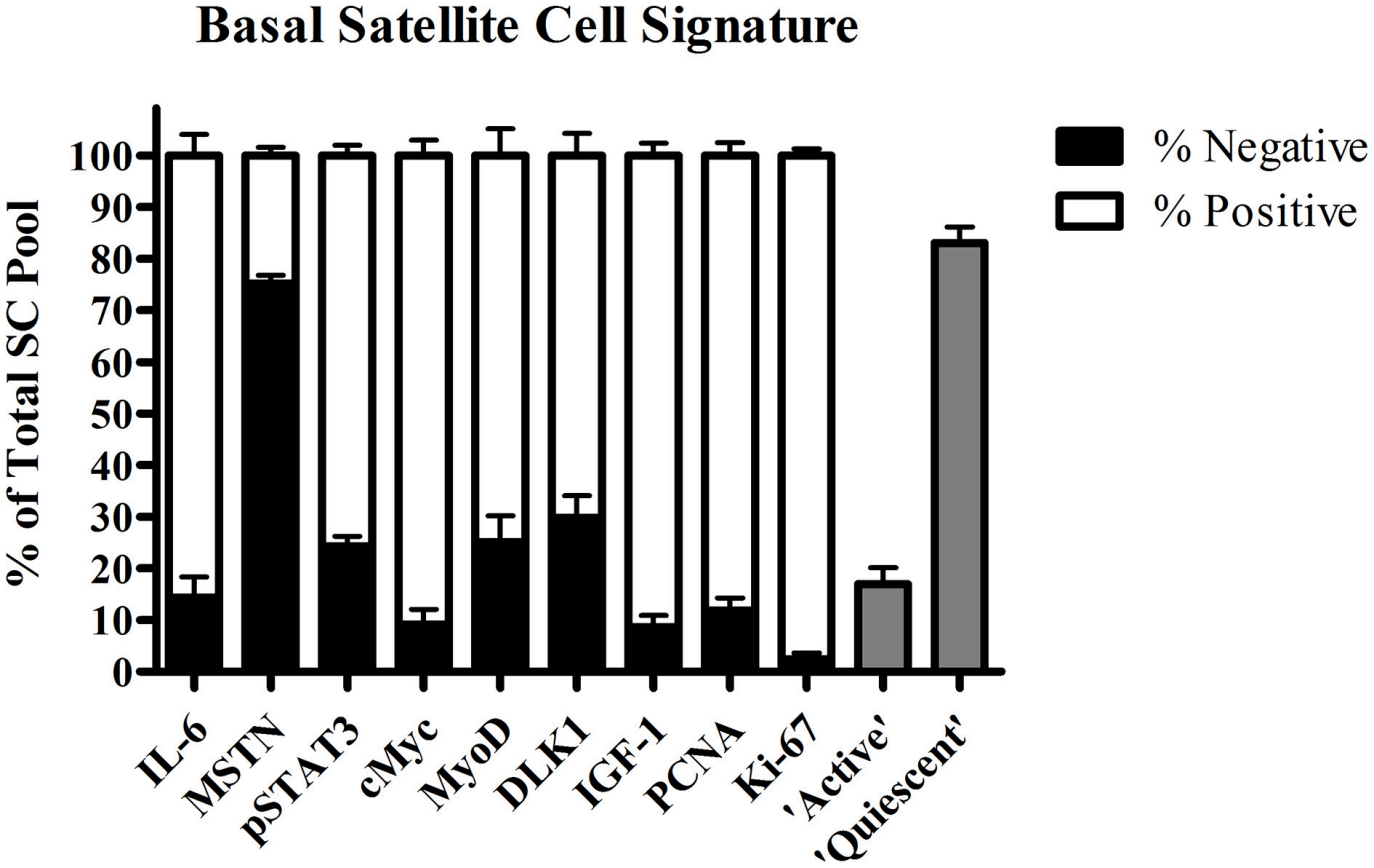
**Proportion (±SEM) of satellite cell pool positive for interleukin-6 (IL-6), myostatin (MSTN), phosphorylated signal transducer and activator of transcription 3 (pSTAT3), cMyc, Myogenic Differentiation (MyoD), Delta Like 1 (DLK1), Proliferating cell nuclear antigen (PCNA), Ki-67, determined by immunohistochemistry, and “active” (G2/M phase) and “quiescent” (G0/G1 phase) satellite cells assessed by flow cytometric in resting ***vastus lateralis*** muscle from healthy young men (combined data from O'Reilly et al., 2008; McKay et al., 2008, 2009, 2010, 2012, 2013; Toth et al., 2011; Snijders et al., 2012, 2014b,c; Cermak et al., 2013; Bellamy et al., 2014)**.

Over the last decade, a genuine effort has been made to further elucidate the factors that control satellite cell function in human skeletal muscle. The co-localization of numerous molecular markers with satellite cells during muscle fiber repair and remodeling has provided us with crucial knowledge on the underlying mechanisms of satellite cell function in healthy and more compromised populations. Subsequently, we were able to construct a (partial) “signature” of a quiescent skeletal muscle satellite cell in healthy young men based on immunofluorescent and flow cytometric analysis (Figure 1). Next, we will discuss the various factors proposed to play an important role in satellite cell activation, proliferation, and differentiation in human skeletal muscle.

## The acute satellite cell response

**Figure 2:**
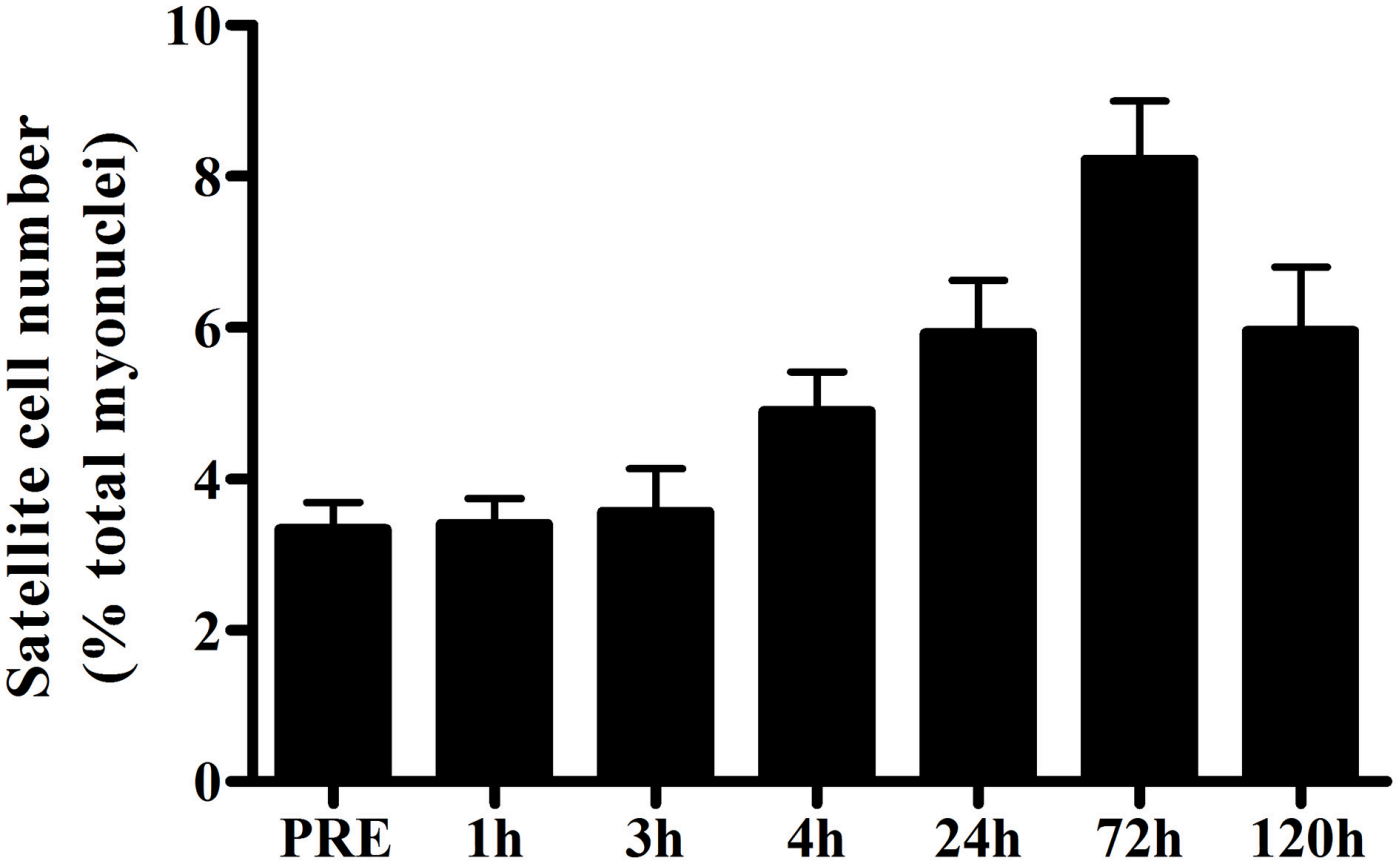
**Mean (±SEM) number of satellite cells (mixed muscle) expressed as a percentage of total myonuclei before and 1, 3, 4, 24, 72, and 120 h after a single bout of eccentric exercise in healthy young men (***n*** = 52; combined data from McKay et al., 2008, 2009, 2010; Toth et al., 2011)**.

In humans, the skeletal muscle adaptive response after a single bout of exercise is the most frequently used model to evaluate the regulation of satellite cell function. In this model, volunteers perform a single bout of exercise and percutaneous muscle biopsies are collected before and at a single or multiple time points during the hours/days of post-exercise recovery. Various modes of exercise are used to investigate different functions of human satellite cells during muscle fiber adaptation. A single bout of unaccustomed eccentric exercise (e.g., muscle lengthening contractions) is the most widely used model to induce (ultra) structural damage to determine the role of satellite cells in muscle fiber repair. From various animal studies it has been well-established that skeletal muscle satellite cells are essential for muscle fiber repair and/or regeneration (Lepper et al., 2011; McCarthy et al., 2011; Murphy et al., 2011; Sambasivan et al., 2011). Likewise, in human skeletal muscle, robust changes in satellite cell number are typically observed in the first few days after performing a single bout of eccentric exercise (Crameri et al., 2004; Dreyer et al., 2006; McKay et al., 2008, 2009; O'Reilly et al., 2008; Mikkelsen et al., 2009; Toth et al., 2011; Cermak et al., 2013; Farup et al., 2014a; see Table 1). By combining various data sets from previous studies from our lab (McKay et al., 2008, 2009, 2010; Toth et al., 2011) we constructed a comprehensive timeline on the change in whole muscle satellite cell number during the first 5 days after a single bout of eccentric exercise in healthy young men (*n* = 52; Figure 2). An increase in satellite cell number becomes apparent at 24 h, and peaks at 72 h of post-exercise recovery. Although most studies have reported the changes in satellite cell content in whole muscle (i.e., in a non fiber type-specific manner), high-force eccentric exercise is generally associated with more selective recruitment of type II muscle fibers (Nardone and Schieppati, 1988; Nardone et al., 1989), and/or type II muscle fiber damage (Friden et al., 1983; Vijayan et al., 2001). Accordingly, Cermak et al. (2013) reported that satellite cell number increased specifically in the type II muscle fibers after performing 300 eccentric contractions in healthy young men. This shows that satellite cells are able to respond in a fiber type specific manner.

**Table 1:** **Change in satellite cell content in response to a single bout of eccentric (damaging) exercise**.

**Publication**	**Sex**	**Age**	***N***	**Exercise protocol**	**Fiber type**	**Pre**	**1 h**	**3 h**	**4 h**	**24 h**	**48 h**	**72 h**	**96 h**	**120 h**	**196 h**
Cermak et al., 2013	M	23 ± 1	8	10 sets 30 reps at −180 deg/s	Mixed	0.091				25%					
					I	0.093				0%					
					II	0.085				73%^*^					
Crameri et al., 2004	M	25 ± 3	8	50 one legged drop down jumps 8 sets 10 reps at −30 deg/s 8 sets 10 reps at −120 deg/s	Mixed	NA					146%		192%^*^		168%^*^
Dreyer et al., 2006	M	23–35	10	6 sets 16 reps at −60 deg/s	Mixed	0.070				141%^*^					
	M	60–75	10	6 sets 16 reps at −60 deg/s	Mixed	0.070				51%^*^					
Hyldahl et al., 2014	M	23 ± 2	7	196 at reps at −180 deg/s	Mixed	0.101				25%					
					I	0.080				30%					
					II	0.106				25%					
McKay et al., 2009	M	22 ± 1	8	10 sets 30 reps at −180 deg/s	Mixed	0.147			73%	155%^*^		185%^*^		108%^*^	
McKay et al., 2010	M	21 ± 2	12	10 sets 30 reps at −180 deg/s	Mixed	0.133				36%^*^					
Mikkelsen et al., 2009	M	23 ± 3	8	200 reps at −120 deg/s	Mixed	0.070									96%^*^
O'Reilly et al., 2008	M	21 ± 2	8	10 sets 30 reps at −180 deg/s	Mixed	0.057			5%	138%^*^		148%^*^		119%^*^	
Toth et al., 2011	M	21 ± 2	12	10 sets 30 reps at −180 deg/s	Mixed	0.155	15%	17%		27%^*^					

This table provides an overview of studies that have assessed the change in muscle fiber satellite cell content in response to a single bout of eccentric (damaging) exercise in both young and older adults. Type I, type I muscle fibers; Type II, type II muscle fibers; Mixed, mixed muscle fibers. Pre value is the average number of satellite cells reported per muscle fiber.

* *Significantly different compared to baseline value*.

**Figure 3:**
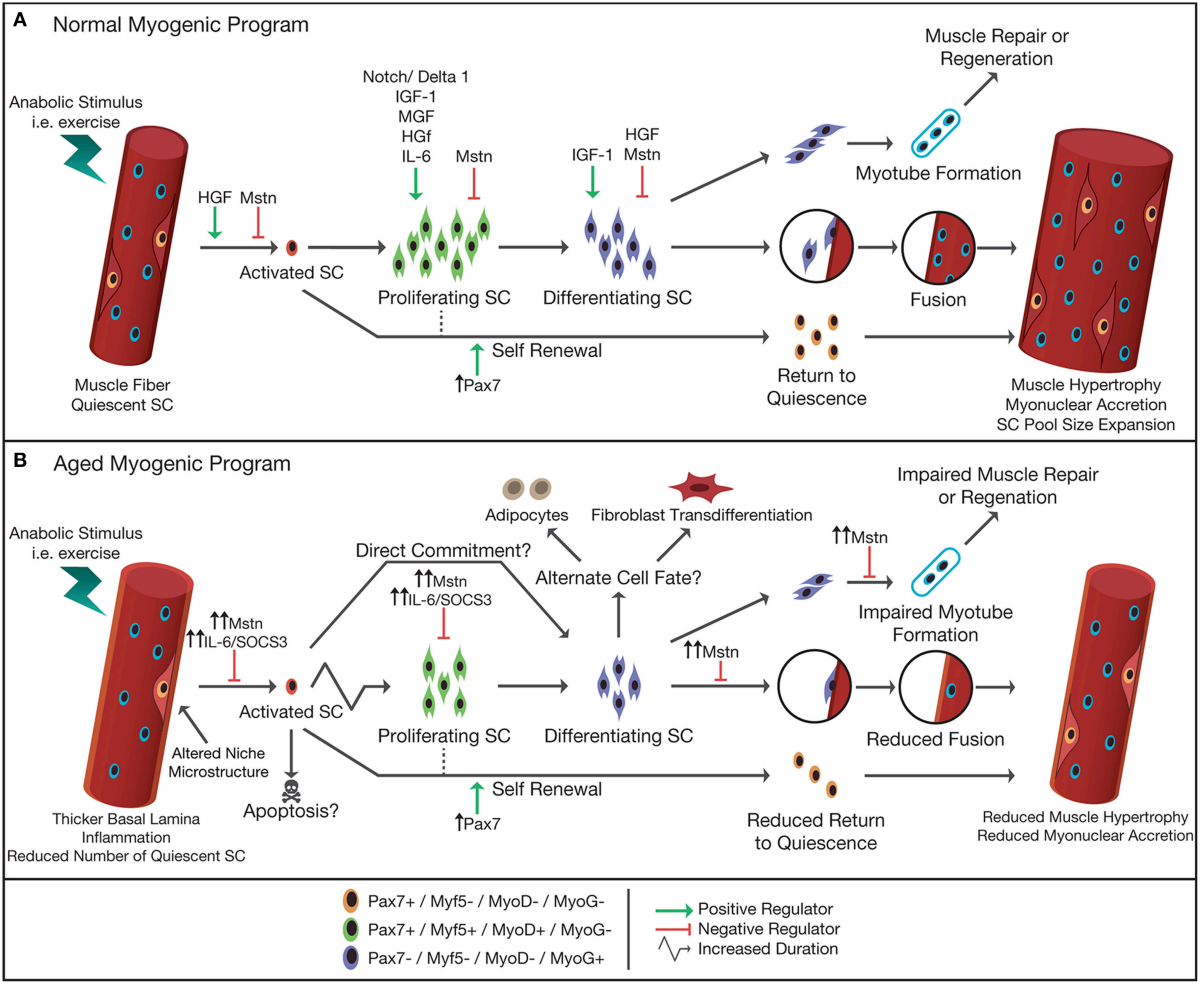
**Schematic representation of normal (A) and aged (B) myogenic program in response to an anabolic stimulus**. In adult skeletal muscle, satellite cells are typically in a quiescent state and reside in a niche between the sarcolemma and basal lamina of their associated muscle fiber. Upon stimulation, i.e., following exercise, satellite cells become activated, and start to proliferate. Following proliferation, satellite cells differentiate, and either fuse with each other forming new myofibers, fuse to an existing muscle fiber donating their nucleus to the fiber thereby allowing muscle fiber hypertrophy, or return back to their quiescent state (self-renewal). The progression of the satellite cell through the myogenic program is orchestrated by the up- or down-regulation of the paired box transcription factor Pax7 and the myogenic regulatory factors (e.g., Myf5, MyoD, MRF4, and Myogenin). A number of factors [e.g., hepatocyte growth factor (HGF), myostatin (Mstn), Notch/Delta1, interleukin-6 (IL-6), mechano growth factor (MGF), and insulin like growth factor-1 (IGF-1)] have been identified to have a positive/negative influence on the different stages of the myogenic program. In aged skeletal muscle the number of muscle satellite cells is reduced and the microstructure of the niche is altered. An increased subclinical level of inflammation and increased Mstn in the circulation has been suggested to impair or delay the proliferative drive of satellite cells in response to anabolic stimuli. Alternatively, it has been hypothesized that in aged muscle the activated satellite cells may also commit directly to differentiation, i.e., skipping the proliferation phase. Studies suggest that aged satellite cells are more likely to differentiate to an alternative cell fate (e.g., adipocytes, fibroblasts) or are directed toward apoptosis, thereby reducing the number of myonuclei formed to allow adequate repair and/or hypertrophy of the muscle fiber. Increased systemic levels of Mstn reduces the fusion of newly formed myonuclei, impairing muscle repair and regeneration, and impairs fusion of myonuclei to existing muscle fibers, limiting muscle fiber growth in aged skeletal muscle.

Satellite cell function can also be evaluated in response to non-damaging exercise (e.g., single session of resistance, endurance, or combined exercise). Likewise, we (McKay et al., 2012, 2013; Snijders et al., 2014b,c) as well as others (Mackey et al., 2007b; Walker et al., 2012; Wernbom et al., 2013) have reported large changes in satellite cell content in response to a single bout of resistance or combined exercise in humans (see Tables 2, 3). Given the substantial expansion of the satellite cell population in the immediate post-exercise recovery period, the single-bout-of-exercise model has been routinely used to evaluate potential factors important in directing satellite cells through the myogenic program. A number of growth factors and inflammatory cytokines released from skeletal muscle, surrounding cells and/or other tissues following exercise have been suggested to play a crucial role in satellite cell activation, proliferation, and/or differentiation during muscle fiber repair and remodeling in humans (Figure 3).

**Table 2:** **Change in satellite cell content in response to a single bout of resistance (non-damaging) exercise**.

**Publication**	**Sex**	**Age**	***N***	**Exercise protocol**	**Fiber type**	**Pre**	**3 h**	**6 h**	**12 h**	**24 h**	**48 h**	**72 h**
Bellamy et al., 2014	M	18–35	23	4 sets 8 reps LP-LE-CP-LC (80% 1 RM)	I	0.107				22%		17%
					II	0.113				34%		31%^*^
Hyldahl et al., 2014	M	24 ± 1	7	351 concentric contractions at reps at 60 deg/s	Mixed	0.099				3%		
					I	0.107				−11%		
					II	0.100				17%		
McKay et al., 2012	M	21 ± 3	10	4 sets 8 reps LE-LP (80% 1 RM)	I	0.059				44%	46%^*^	
					II	0.095				26%	44%^*^	
	M	70 ± 4	10	4 sets 8 reps LE-LP (80% 1 RM)	I	0.060				10%	62%	
					II	0.050				34%	6%	
McKay et al., 2013	M	21 ± 3	10	4 sets 8 reps LE-LP (80% 1 RM)	Mixed	0.138	5%			20%^*^	33%^*^	
	M	70 ± 4	10	4 sets 8 reps LE-LP (80% 1 RM)	Mixed	0.092	–1%			15%	29%	
Snijders et al., 2014c	M	22 ± 1	10	10 sets 8 reps LE-LP (80% 1 RM)	I	0.094			14%^*^	19%	42%^*^	49%^*^
					II	0.102			1 %	14%	34%^*^	48%^*^
	M	73 ± 1	10	10 sets 8 reps LE-LP (80% 1 RM)	I	0.078			10%^*^	22%	40%^*^	45%^*^
					II	0.077			−5 %	1%	8%	34%^*^
Walker et al., 2012	M	27 ± 2	5	8 sets 10 reps LE (70% 1 RM)	Mixed	0.068		−13%		157%^*^		
	M	70 ± 2	6	8 sets 10 reps LE (70% 1 RM)	Mixed	0.062		−1%		6%		
	F	27 ± 2	5	8 sets 10 reps LE (70% 1 RM)	Mixed	0.051		7%		21%		
	F	70 ± 2	5	8 sets 10 reps LE (70% 1 RM)	Mixed	0.032		115%		146%		

This table provides an overview of studies that have assessed the change in muscle fiber satellite cell content in response to a single bout of resistance (non-damaging) exercise in both young and older adults. Type I, type I muscle fibers; Type II, type II muscle fibers; Mixed, mixed muscle fibers; LP, leg press; LE, leg extension; LC, leg curl; CR, calf raises. Pre value is the average number of satellite cells reported per muscle fiber.

* *Significantly different compared to baseline value*.

**Table 3:** **Change in satellite cell content in response to a single bout of various other exercise modalities**.

**Publication**	**Sex**	**Age**	***N***	**Exercise protocol**	**Fiber type**	**Pre**	**Post**	**1 h**	**9 h**	**24 h**	**48 h**	**192 h**
**COMBINED EXERCISE**												
(Snijders et al., 2012)	M	20 ± 1	8	4 × 5 min 65% Wmax + 4 × 45% Wmax Whole-body RT (65% 1 RM)	I	0.080	3%		26%			
					II	0.066	9%		24%			
**BLOOD FLOW RESTRICTED EXERCISE**												
(Wernbom et al., 2013)	M and F	22 ± 3	6 vs. 1	5 sets until failure (30% 1 RM)	Mixed	0.200		36%^*^		±43%^*^	±48%^*^	
**ENDURANCE EXERCISE**												
(Mackey et al., 2007b)	M	25 ± 3		36 km run	Mixed	NA						27%^*^

This table provides an overview of studies that have assessed the change in muscle fiber satellite cell content in response to a single bout of various other exercise modalities. Type I, type I muscle fibers; Type II, type II muscle fibers; Mixed, mixed muscle fibers. Pre value is the average number of satellite cells reported per muscle fiber.

* *Significantly different compared to baseline value*.

## Satellite cell regulators:

### Insulin-like growth factor-1 (IGF-1)

IGF-1 is a circulating growth factor that is produced and released systemically by the liver and locally by the muscle. Three spliced variants of IGF-1 have been described in skeletal muscle (i.e., IGF-1Ea, IGF-1Eb, and IGF-1Ec) and each has been hypothesized to contribute to some extent to muscle fiber regeneration (Rotwein et al., 1986; Yang et al., 1996; Chakravarthy et al., 2000). *In vitro* work has demonstrated that C2C12 myoblasts proliferate when exposed to either IGF-1Ec [also known as mechano growth factor (MGF)], or IGF-1Ea (Yang and Goldspink, 2002). In contrast, C2C12 myoblasts show impaired differentiation to MGF, but not with exposure to IGF-1Ea (Yang and Goldspink, 2002). Consistent with these results, animal studies have reported that MGF mRNA expression in muscle increases as early as 24 h after mechanical damage, however, IGF-1Ea is only elevated after 5–10 days of recovery (Hill and Goldspink, 2003; Hill et al., 2003). Similarly, muscle MGF mRNA expression has been shown to be increased significantly, with no changes in IGF-1Ea mRNA expression, approximately 2 h after performing eccentric cycle ergometry in healthy, young men (Hameed et al., 2008). Hill et al. (2003) suggest that MGF is related to the activation of satellite cells, while IGF-1Ea is related to an increased need for muscle protein synthesis in the late phase of satellite cell differentiation. Recent work from our lab in healthy, young men supports this notion. We have observed a strong temporal relationship between MGF and Myf5, a known driver of satellite cell proliferation, mRNA abundance in whole skeletal muscle during recovery from eccentric exercise (McKay et al., 2008). In contrast, we found that IGF-1Ea expression was strongly correlated with Mrf4 expression, a MRF known for its role in satellite cell differentiation (McKay et al., 2008). This was later confirmed in human muscle tissue at the protein level (Philippou et al., 2009), further supporting the temporal association of IGF-1 splice variants with the myogenic program. Interestingly, Hellsten et al. (1996) have reported an increase in the number of IGF-1^+^ cells located in the satellite cell position after 7 days of intense military training, which included 150 km of terrain marching carrying a 30-kg load. However, as no co-localization with a satellite cell marker was performed, the authors could not exclude that a proportion of these “spindle shaped IGF-1 immunoreactive cells” may have been activated fibroblasts or inflammatory cells. With the use of a pan-IGF-1 antibody we have more recently been able to show that IGF-1 can indeed co-localize with Pax7^+^ satellite cells in human muscle cross-sections (Grubb et al., 2014). Furthermore, whereas no IGF-1 and Pax7 co-localization was observed in resting muscle biopsy samples, the majority of satellite cells were reported to be IGF-1^+^ 24 h after 300 high-velocity maximal eccentric contractions (McKay et al., 2008). This is further supported and consistent with the timing of the observed change in IGF-1 splice variant mRNA expression after eccentric exercise in healthy, young men (Bamman et al., 2001; Hameed et al., 2008; McKay et al., 2008). Overall, both animal and human studies provide substantial evidence that IGF-1 plays an important role in skeletal muscle fiber repair which may, in part, include satellite cell activation, proliferation, and differentiation.

### Hepatocyte growth factor (HGF)

HGF is a stromal mesenchymal-derived growth factor initially named for its mitogenic properties in the liver. In response to tissue damage, HGF is activated in the cytosol through cleavage of its pro-peptide serine protease HGF activator (HGFA) (Miyazawa et al., 1996; Tjin et al., 2004), which in itself is activated from its zymogen form (Shimomura et al., 1993). HGF is known to activate quiescent satellite cells by binding to its receptor c-Met, which is localized to the satellite cell membrane (Shimomura et al., 1993; Allen et al., 1995; Tatsumi et al., 1998; Yamada et al., 2010). Administration of recombinant HGF into animal muscle results in an increase in the number of BrdU^+^ or MyoD^+^ satellite cells and a reduction in muscle fiber formation (Tatsumi et al., 1998; Miller et al., 2000), which supports the notion that HGF is a potent stimulator of satellite cell activation and proliferation, but not differentiation (Allen et al., 1995; Anastasi et al., 1997; Gal-Levi et al., 1998; Tatsumi et al., 1998, 2006a; Miller et al., 2000; Li et al., 2009). In addition, it has been demonstrated that when myoblasts are exposed to stretch, HGF is released from the extracellular tethering, suggesting an autocrine/paracrine function of HGF (Tatsumi et al., 2001, 2002, 2006a,b; Yamada et al., 2010). In contrast, there is very little data on the role of HGF in satellite cell function during repair/regeneration in human skeletal muscle. To our knowledge, only one study has assessed HGF in relation to satellite cell function during muscle fiber repair in humans. In this study we reported a substantial increase in satellite cell number accompanied by a concomitant increase in HGFA protein content 24 h following a single bout of eccentric exercise in healthy young men (O'Reilly et al., 2008). In addition, an (non-significant) increase in active HGF was observed during the first 24 h of post-exercise recovery. Interestingly, in the same study we found that serum HGF was significantly elevated at 4 h after the muscle damage protocol (O'Reilly et al., 2008). Although the rise of HGF in circulation gives no information as to the origin of HGF, it is conceivable that the increase in serum HGF represents a systemic response (non-muscular) to localized muscle damage. This would be consistent with experiments in rats demonstrating that HGF mRNA and protein expression are upregulated in non-muscle tissue following injury of skeletal muscle (Suzuki et al., 2002). Furthermore, HGF may be carried to the site of injury by granulocytes, known to increase in number during the inflammatory response to muscle fiber damage (Ohnishi et al., 2006). On the other hand, HGF levels may also rise simply as a result of the muscle damage itself, as proteins may leak into circulation by disruption of skeletal muscle fiber membranes. Future studies should aim to further establish the origin of circulating HGF levels following muscle fiber damage and its precise role in satellite cell function in human skeletal muscle.

### Interleukin 6 (IL-6)

Traditionally, Interleukin 6 (IL-6) was considered to be an inflammatory cytokine (Kishimoto, 2005). However, it is well-established that IL-6 is also produced by skeletal muscle in response to exercise (see also review Pedersen and Febbraio, 2008). As such, IL-6 is also referred to as a “myokine” (Keller et al., 2003; Pedersen and Febbraio, 2008). An exercise induced increase in circulating levels of IL-6 has been observed to enhance fat-oxidation, improve insulin-stimulated glucose uptake, and at low levels has an anti-inflammatory effect (Pedersen and Febbraio, 2008). In addition, animal studies have suggested that IL-6 may also play a significant role in satellite cell mediated muscle fiber growth. IL-6 knock-out mice show a blunted muscle fiber hypertrophic response and less satellite cell mediated myonuclear accretion compared to wild-type mice following overload induced muscle hypertrophy (Serrano et al., 2008). In addition, it was reported that satellite cells from IL-6 knock-out mice have a reduced proliferative capacity, both *in vitro* as well as *in vivo*, which was shown to be related to a loss of IL-6 mediated signal transducer and activator of transcription 3 (STAT3) signaling (Serrano et al., 2008). STAT3 is a downstream target of IL-6 (Levy and Lee, 2002; Trenerry et al., 2008), and upon IL-6 binding to the IL-6Rα receptor, JAK2 is phosphorylated and initiates the phosphorylation of STAT3. This leads to the subsequent homodimerization and translocation of pSTAT3 to the nucleus (Rawlings et al., 2004). In the nucleus, pSTAT3 binds to the γ-interferon activation sequence (GAS) element where it promotes the transcription of downstream genes that are responsible for a variety of cellular functions including proliferation, migration and the prevention of apoptosis (Ivanova et al., 2004; Serrano et al., 2008). STAT3 is known to regulate a number of its upstream signaling cascade members including IL-6, GP130, IL-6Rα, and suppressor of cytokine signaling 3 (SOCS3). The STAT3 pathway is regulated in a negative feedback loop through interaction with JAK2. SOCS3 can bind phosphotyrosines on JAK2 and physically block STAT3 from binding to JAK2. In addition, SOCS3 can recruit ubiquitin-transferases leading to the ubiquitination and degradation of JAK2 (Rawlings et al., 2004). Previously, we have shown that the IL-6Rα receptor is expressed on human skeletal muscle satellite cells (McKay et al., 2009, 2013; Toth et al., 2011). Whereas, IL-6 is virtually non-detectable in the resting/undamaged state, we have shown a substantial rise in the proportion of satellite cells positive for IL-6 (approximately 60–80%) as early as 3 and up to 24 h after a single bout of eccentric exercise in healthy young men (McKay et al., 2009; Toth et al., 2011). Interestingly, in these studies, satellite cell content increased significantly at 24 h and peaked 72 h following exercise, at which time the number of IL-6^+^ satellite cells had already decreased to baseline levels (McKay et al., 2009; Toth et al., 2011). In addition, Toth et al. (2011) reported a significant increase in the number of pSTAT3^+^ satellite cells 1, 3, and 24 h after a single bout of eccentric exercise. Moreover, the increase in the number of pSTAT3^+^ satellite cells was accompanied by a concomitant increase in the number of cMyc^+^/Pax7^+^ cells 24 h post-exercise. cMyc is a critical regulator of the cell cycle, responsible for the transition from G1 to S phase (Dang, 1999) and is upregulated in response to IL-6 signaling (Nabata et al., 1990) in a STAT3 induced GP130 mediated manner (Kiuchi et al., 1999). The early increase in pSTAT3^+^ cells provides temporal evidence that following a bout of damaging exercise, STAT3 is phosphorylated leading to downstream signaling events resulting in the proliferation of human satellite cells. This is in accordance with a recent study in animals showing that conditional ablation of STAT3 in satellite cells resulted in an expansion of the satellite cell pool size during regeneration, but comprised myogenic differentiation, preventing the contribution of these cells to regenerating myofibers (Tierney et al., 2014). Thus, it appears that IL-6 is a critical signaling molecule, contributing to the coordination of satellite cell function *in vivo*. This theory is further supported by data from human studies that report an inhibition of the satellite cell response to exercise-induced muscle damage via the administration of non-steroidal anti-inflammatory drugs (NSAID) (Mackey et al., 2007b; Mikkelsen et al., 2009), which block the inflammatory cascades responsible for cytokine production. Furthermore, the temporal nature of the IL-6 response has been suggested to be essential for an appropriate satellite cell response. Following exercise, IL-6 is systemically released in a temporal manner, increasing rapidly and remaining elevated for a few hours to a few days (Serrano et al., 2008; McKay et al., 2009). This temporal increase in IL-6 appears to have a positive impact on muscle health, augmenting glucose uptake and muscle stem cell function. A chronic elevation of circulating IL-6 observed in disease states appears to be detrimental, impairing muscle protein synthesis, and contributing to the loss of skeletal muscle mass (Haddad et al., 2005). In addition to the temporal expression of IL-6, the relative local concentration of IL-6 also appears to be of great importance, dictating the action of IL-6 on the target tissue. High local concentrations such as those observed in the satellite cell compartment (or niche) following damaging exercise may confer a positive effect on these cells, promoting the induction of the cell-cycle through the targeted up-regulation of cMyc and cyclin D1 (McKay et al., 2009). While a relatively modest temporal increase in IL-6 in the circulation (presumably released from the working muscle) may serve to sensitize the muscle to IL-6 by increasing both IL-6Rα and GP130 mRNA, a chronic elevation IL-6 may be detrimental to the same pathway (McKay et al., 2009). Therefore, the local concentration of IL-6 in the vicinity of the satellite cell niche may be of more relevant for the satellite cell response than the systemic concentration of IL-6. This concept of compartmentalization of the IL-6 response may contribute to the pleiotropic effect of IL-6 on skeletal muscle.

### Myostatin

Myostatin is a member of the transforming growth factor-β (TGF-β) superfamily and acts as a potent negative regulator of skeletal muscle growth (McPherron et al., 1997; McPherron and Lee, 2002). McPherron et al. (1997) were the first to characterize the phenotypic response to a myostatin gene mutation in mice. They showed that inhibition or mutation of the myostatin gene resulted in mice with 2–3 fold greater muscle mass as compared to wild-type mice (McPherron et al., 1997). This initial study was soon followed by other reports that naturally occurring myostatin mutations are accompanied by excessive muscle fiber hypertrophy/hyperplasia as observed in double-muscled cattle, sheep and dogs (Grobet et al., 1997; Kambadur et al., 1997; Clop et al., 2006; Mosher et al., 2007). In humans, a single case study has been published in which a new born was identified as a carrier of a myostatin null mutation, after being noted to have an unusual amount of muscle mass at birth (Schuelke et al., 2004). Multiple mechanistic pathways through which myostatin is able to regulate skeletal muscle growth have already been identified. Myostatin has been shown to induce muscle wasting by acting on the ubiquitin proteolytic system, evidenced by the myostatin induced up-regulation of genes like atrogin-1, FoxO1, and MuRF-1 (McFarlane et al., 2006). Secondly, myostatin is known to affect muscle mass by regulating myogenesis. *In vitro* experiments have shown that myostatin blocks myoblast proliferation (Thomas et al., 2000; Taylor et al., 2001) and differentiation by the down regulation of MyoD (Langley et al., 2002) and up-regulation of p21, a regulator of cell cycle progression (McFarlane et al., 2011). In addition, myostatin inhibits satellite cell proliferation and self-renewal (McCroskery et al., 2003). In contrast, *in vivo* animal models have reported discrepant findings. Whereas, some do (McCroskery et al., 2003; McFarlane et al., 2011) others do not (Amthor et al., 2009; Wang and McPherron, 2012) show that satellite cell proliferation and pool size are significantly higher in muscle tissue from myostatin knock-out compared with wild-type mice. In addition, pharmacological inhibition of the myostatin/activin A pathway has been demonstrated to induce muscle hypertrophy with little to no fusion of satellite cells to the myofibers (Lee et al., 2012). These *in vitro* and animal studies do not, however, address the potential role of myostatin during muscle fiber growth in a more physiological scenario, such as in response to a single bout of exercise or during adaptation to prolonged exercise training in humans. We have previously shown that myostatin is expressed in human skeletal muscle satellite cells (McKay et al., 2012; Snijders et al., 2014b,c). During resting conditions approximately 80% of the satellite cell pool is co-localized with myostatin. In addition, by the utilization of the single-bout-exercise model we were able to show that the number of myostatin^+^ satellite cells in both type I and type II muscle fibers decreases substantially, the timing of which is consistent with an increase in the number of MyoD^+^ satellite cells and number of satellite cells in the S-phase of the cell cycle (McKay et al., 2012; Snijders et al., 2014b,c). Together, *in vivo* human, animal and cell studies suggest that myostatin is a powerful negative regulator of muscle growth and is therefore possibly critical in the muscle fiber adaptive response to a stimulus like exercise training. However, whether this effect is, at least in part, regulated through muscle satellite cells remains to be further elucidated.

## Satellite cells and muscle fiber hypertrophy

Although it has been well-established from both animal and human studies that satellite cells are essential in muscle fiber repair and regeneration, there is still an ongoing debate on the importance of satellite cells during muscle fiber hypertrophy. Early γ-irradiation studies suggested that muscle fiber growth in mice is virtually non-existent when satellite cells are ablated (Rosenblatt and Parry, 1992, 1993). However, it was later argued that the cellular specificity of γ-irradiation was too low to truly assess the absolute requirement of satellite cells in overload induced muscle fiber hypertrophy (McCarthy et al., 2011). In response, McCarthy et al. (2011) used a mouse model to conditionally and specifically ablate Pax7^+^ cells to re-evaluate the hypothesis that satellite cells are required to support skeletal muscle hypertrophy. They reported that in normal, non-satellite cell depleted muscle (i.e., sham condition), overload induced muscle fiber hypertrophy was accompanied by the contribution of new myonuclei from satellite cells (McCarthy et al., 2011). However, muscle fiber hypertrophy (during 2 weeks of overload) was found to be similar in the satellite cell ablated condition, in which satellite cells were depleted by greater than 90% (McCarthy et al., 2011). As such, the authors concluded that satellite cells are not essential to support hypertrophy in mouse skeletal muscle (McCarthy et al., 2011). However, Fry et al. (2014b) observed that exposing these genetically modified mice to prolonged overload (8 weeks) compromises muscle fiber hypertrophy. In addition, the authors suggested that the substantial increase in extracellular matrix during this period of overload may contribute to the impaired muscle fiber growth response (Fry et al., 2014b). Taken together, these data indicate that under specific experimental conditions, the existing myonuclei have the intrinsic ability to increase their capacity to such an extent that additional myonuclei are not required to support muscle fiber growth but this growth appears to be unsustainable. In other words, satellite cells do appear to be required to support extensive muscle fiber hypertrophy in response to an anabolic stimulus (Fry et al., 2014b). Although these studies address an intriguing question in an experimental (non-physiological) model of muscle biology, translating these results to human physiology remains a challenge. The reality is that these experimental models are important as they provide proof of principle for biological phenomena. They do not, however, establish that these principles are relevant under physiological conditions. Most human studies are descriptive, demonstrating correlations between changes in myonuclear content, satellite cell number, and muscle fiber size in response to an anabolic stimulus. Nonetheless, such studies are crucial in understanding the role of satellite cell content and function in skeletal muscle fiber hypertrophy in an *in vivo* human setting. A growing number of studies have now consistently demonstrated that muscle fiber hypertrophy is accompanied by a concomitant increase in satellite cell and/or myonuclear content during prolonged resistance type exercise training (Kadi and Thornell, 2000; Kadi et al., 2004c; Olsen et al., 2006; Petrella et al., 2006; Mackey et al., 2007a, 2011a,b; Verney et al., 2008; Verdijk et al., 2009, 2014; Menon et al., 2012; Leenders et al., 2013; Suetta et al., 2013; Farup et al., 2014b). In addition, a positive correlation between the increase in muscle fiber size and increase in satellite cell content in response to prolonged resistance type exercise training in healthy young and elderly men and women has been well-established (Petrella et al., 2006; Verdijk et al., 2010, 2014; Mackey et al., 2011b; Bellamy et al., 2014). This would suggest that satellite cells do play an important role in muscle fiber hypertrophy in response to a growth stimulus in human muscle, irrespective of whether they are “required” or not. Interestingly, Bellamy et al. (2014) have recently shown that the acute changes in satellite cell content in response to a single bout of resistance type exercise are related to long-term changes in skeletal muscle mass and cross-sectional area following 16 weeks of resistance type exercise training in healthy, young men.

Alternatively, we have recently hypothesized that satellite cells may also play an important role in non-hypertrophic skeletal muscle remodeling (Joanisse et al., 2013, 2015). We have demonstrated that 6 weeks of high intensity sprint interval or moderate intensity continuous aerobic exercise does not result in changes in type I or type II muscle fiber size and/or satellite cell content in untrained healthy young men and women (Joanisse et al., 2013, 2015). Interestingly however, the number of proliferating satellite cells increased significantly in type I and type II muscle fibers in response to both exercise programs (Joanisse et al., 2015). Furthermore, a significant increase in the percentage of hybrid fibers (i.e., fibers that are co-expressing both myosin heavy chain I and II) after high intensity sprint interval training was accompanied by a significant increase in the number of satellite cells associated with these hybrid fibers (Joanisse et al., 2013). This increase in the number of satellite cells associated with hybrid fibers following training may be required in order for fast-to-slow muscle fiber transitions to occur in response to prolonged exercise training. However, others have shown that a shift in fiber type distribution can occur independent of changes in satellite cell content following 12 weeks of aerobic training in healthy young men (Fry et al., 2014a). In addition, several animal studies have reported that muscle fiber type transition can occur in the absence of satellite cell activation (Rosenblatt and Parry, 1993; Adams et al., 2002; Fry et al., 2014b). Differences between studies may be explained by the different models (i.e., exercise modality, volume, and/or intensity) used to evaluate satellite cell function in muscle fiber adaptation. The precise role of satellite cells in non-hypertrophic skeletal muscle remodeling remains largely to be elucidated. It does, however, open a wide range of new research questions on the role of satellite cells in skeletal muscle remodeling.

Taken together, evidence from human studies strongly suggests that satellite cells play a key role in skeletal muscle fiber hypertrophy in humans. The premise that satellite cells may also play an important role in other processes of skeletal muscle fiber remodeling is thought-provoking and warrants future studies. In addition, more *in vivo* human studies are needed to further decode the factors that trigger satellite cells from quiescence to their activated state. As impairments in satellite cell activation have been hypothesized to play a key role in the blunted skeletal muscle fiber growth response after a stimulus like exercise training in healthy elderly and/or more clinically compromised populations, the identification of these specific factors may prove to be paramount in the potential development of novel intervention strategies to more effectively combat the loss of muscle mass associated with aging and/or disease.

## Skeletal muscle aging

Aging is accompanied by a progressive loss of skeletal muscle mass, also known as sarcopenia. At the muscle fiber level, this loss of muscle mass is mainly characterized by type II muscle fiber atrophy. In addition, we (Verdijk et al., 2009, 2010, 2012, 2014; McKay et al., 2012, 2013; Leenders et al., 2013) as well as others (Verney et al., 2008; Carlson et al., 2009; Suetta et al., 2013; Mackey et al., 2014) have consistently shown that type II muscle fiber atrophy in aged muscle is accompanied by a type II muscle fiber type specific reduction in satellite cell content. Although satellite cells are considered to be the main (or only) source of new myonuclei, whether they are a key regulator of skeletal muscle mass maintenance throughout life remains debatable. Based on a study in animals showing that a decline in satellite cell content precedes age-related muscle fiber atrophy (Brack et al., 2005) and the observation that satellite cell content has been shown to be a strong predictor of muscle fiber size in older adults (Verdijk et al., 2010), it has been hypothesized that an age-related decline in type II muscle fiber satellite cell content may lead to a reduced capacity for muscle fiber maintenance, leading to specific type II muscle fiber atrophy typically observed in aged muscle (Snijders et al., 2009). This notion has, however, recently been challenged by work using a conditional model of satellite cell ablation (Fry et al., 2015). In this animal study, ±85% of the satellite cells were depleted from muscle in the experimental group, thereby severely impairing muscle fiber regeneration over the entire life-span as compared to a control group. Following 24 months, significant atrophy was apparent in both the experimental and control animals (Fry et al., 2015). This suggests that the loss of satellite cells through-out adulthood does not accelerate sarcopenia in aging mice (Fry et al., 2015). In addition, no difference was observed in muscle specific force and raw grip strength between the two groups. Yet, satellite cell depletion did result in a significant accumulation of extracellular matrix proteins throughout life (Fry et al., 2015). An important consideration in the interpretation of these results is that the mice were sedentary throughout their lives, which does not represent a normal physiological condition. The fact is that mice living in a cage from birth to death is a level of sedentary behavior that far exceeds a sedentary lifestyle in a human. Moreover, the recovery from major life events, like sickness or injury, which is typically observed throughout life in humans, is not accounted for in this model and has been suggested to be of major importance in the progression of sarcopenia (Baumgartner et al., 1998; Paddon-Jones et al., 2008; Wall et al., 2013). Thus, the translation of these results to real life settings in a human model remains a challenge.

Since it is impossible to monitor a human throughout the entire lifespan, to study the mechanisms underlying the loss of muscle mass with age, studies in humans are mostly restricted to cross-sectional research designs (as discussed above) and/or more short-term models of muscle fiber atrophy. Limb immobilization is a model extensively used, which allows for the assessment of short-term skeletal muscle fiber atrophy in a longitudinal fashion. The use of short term disuse muscle fiber atrophy models may be of great clinical importance as it is likely that the accumulation of short periods of muscle disuse that occur throughout an individual's lifespan may contribute significantly to the etiology of age-related sarcopenia. So far, few studies have assessed the potential role of satellite cells in the development of (disuse) muscle fiber atrophy in humans. Satellite cell pool size has been reported to remain unchanged when muscle fiber atrophy was induced by 5 or 14 days of single leg immobilization in both young (Carlson et al., 2009; Snijders et al., 2014a; Dirks et al., 2014b) and older men (Carlson et al., 2009; Suetta et al., 2013). Others have even reported a significant increase in the number of satellite cells in both type I and type II muscle fibers in response to disuse atrophy in healthy young males (Suetta et al., 2013). In this study by Suetta et al. (2013), 2 weeks of single leg immobilization was followed by 4 weeks of reloading in both young and older men. Type I and type II muscle fiber size returned back to baseline and was accompanied by a significant increase in satellite cell number during the 4 weeks of reloading in young men (Suetta et al., 2013). In contrast, muscle fiber size did not recover and satellite cell content remained low during the reloading period in older men. The authors suggest that a reduced sensitivity to certain growth factors, that also regulate satellite cell function, may explain this impaired recovery from short-term physical inactivity in older men (Suetta et al., 2013). In contrast, animal studies have demonstrated that satellite cells are not essential in the re-growth of skeletal muscle following 2 week of hindlimb suspension (Jackson et al., 2012). In this study, the number of myonuclei remained unchanged during hindlimb suspension and reloading. In other words, myonuclear domain size (i.e., the ratio between muscle fiber size and myonuclear content) was able to contract during short-term muscle fiber atrophy and expands again during reloading making additional myonuclei, and thereby satellite cell activation and/or proliferation, not a requirement. Likewise, in human skeletal muscle, myonuclear number does not appear to be lost in response to short-term (5–14 days) limb immobilization in both young and older men (Dirks et al., 2014a,b; Snijders et al., 2014a). However, mixed results have been reported during aging. Although the vast majority of studies in humans do not observe a loss in myonuclear number during age-related muscle fiber atrophy (Kadi et al., 2004a; Dreyer et al., 2006; Petrella et al., 2006; Verdijk et al., 2007; Mackey et al., 2014; Karlsen et al., 2015), this has been suggested to be mainly related to the lack of fiber type specific data and the number and age of participants included. In the largest (*N* = 152) cross-sectional study of healthy men to date, we have recently shown that type II muscle fiber myonuclear number was not different between healthy young (18–49 years) and older men (50–69 years), but was significantly lower in healthy elderly (70> years) men (Verdijk et al., 2014). Furthermore, it is important to note that most studies report changes in myonuclear number per fiber type as an average of the entire muscle biopsy sample, but, previous animal studies show that loss of myonuclear content may be differentially regulated in small compared with large muscle fibers (Brack et al., 2005). Conversely, a more recent study was not able to replicate these results in human skeletal muscle (Karlsen et al., 2015). The former may, however, be explained by the relative young age (66 ± 4 years) of older men included in this study. It would be interesting to observe whether similar results would be obtained in older, frail and/or more clinical populations.

During more severe pathological conditions of muscle wasting, like Duchenne or myotonic muscular dystrophy, exhaustion of the satellite cell pool has been hypothesized to play an important role in the inability to offset degenerative events in animal models (Decary et al., 2000; Luz et al., 2002; Thornell et al., 2009). Although this hypothesis has been challenged by more recently published cross-sectional data from human muscular dystrophy studies (Kottlors and Kirschner, 2010; Bankole et al., 2013), there is no indication that exhaustion of the satellite cell pool occurs with aging in humans. For example, no difference in skeletal muscle telomere length has been observed between healthy young and elderly men and women (Ponsot et al., 2008). Accordingly, a lower satellite cell pool size in type II muscle fibers, typically observed in human aged muscle, does not prevent muscle fiber hypertrophy in healthy elderly men and women in response to prolonged resistance type exercise training (Mackey et al., 2007a; Verney et al., 2008; Verdijk et al., 2009, 2010, 2014; Leenders et al., 2013). Furthermore, satellite cell content has been shown to be similar between age-matched healthy untrained elderly and masters endurance athletes (Mackey et al., 2014).

Aside from satellite cell content, impairments in satellite cell function have also been suggested to be an important contributing factor to the loss of muscle mass with increasing age (Figure 3). For example, under homeostatic conditions fibroblast growth factor 2 (FGF2) expression is considerably increased in the aged stem cell niche, causing a down-regulation of Sprouty1 in quiescent satellite cells (Chakkalakal et al., 2012). Sprouty1 is a known negative regulator of FGF2 signaling and has previously been shown to be required for the return to quiescence and self-renewal of satellite cells during muscle fiber regeneration (Abou-Khalil and Brack, 2010; Shea et al., 2010). Hence, it has been suggested that the dysregulation of the FGF2/Sprouty1 signaling cascade drives a subset of satellite cells to exit the quiescent state and lose their self-renewal capacity, and thereby diminishing muscle regeneration in aged mouse muscle (Chakkalakal et al., 2012). Likewise, impaired up-regulation of Notch ligand Delta 1 in aged satellite cells impairs satellite cell activation, proliferation, and muscle repair following injury (Conboy et al., 2003, 2005; Wagers and Conboy, 2005). Notch signaling has also been implicated in satellite cell homeostasis, as reduced Notch signaling leads to myogenic differentiation, bypassing self-renewal, eventually resulting in the loss of satellite cells (Bjornson et al., 2012; Mourikis et al., 2012). Furthermore, others have also recently shown that in aged mice, resting satellite cells lose reversible quiescence due to switching to an irreversible dormant state, caused by the de-repression of p16^INK4a^ (Sousa-Victor et al., 2014).

A recent publication in Science reported that systemic levels of growth differentiation factor 11 (GDF11) were substantially reduced with age (Sinha et al., 2014). In the same report dramatic positive effects were observed on satellite cell number, muscle fiber regeneration, size, and function when recombinant GDF11 was administered for 28 days to aged mice (Sinha et al., 2014). These results were rather surprising given the fact that, as a TGF-β family member, GDF11 is highly related to myostatin. The latter which is known to be a direct inhibitor of muscle fiber growth (McPherron et al., 1997; McPherron and Lee, 2002). It was argued that the specificity of the antibody used in this study was too low to discriminate between GDF11 and myostatin. In response, Egerman et al. (2015) established a specific immunoassay to measure only changes in GDF11 protein, and demonstrated that in both animal and humans GDF11 was actually increased with aging. Furthermore, they showed that systemic injection of GDF11 impairs satellite cell proliferation and differentiation, resulting in decreased muscle fiber regeneration in mice. This work appears to be more in line with other previous publication on the inhibitory function of GDF11 on muscle fiber growth and regeneration (Gamer et al., 2001; Souza et al., 2008; Lee and Lee, 2013). Altogether, these studies clearly suggest that specific signaling proteins cause a disruption of satellite cell activation, proliferation, and/or differentiation with aging during muscle fiber repair and remodeling.

Previous animal studies have also shown that extrinsic signals in the satellite cell micro-environment are important in the age-related dysregulation of satellite cells during muscle fiber regeneration. To investigate whether muscle regeneration from old animals could be restored to a more youthful state by environmental exposure, studies have utilized experimental models including heterochronic (young-to-old or old-to-young) tissue transplantation and heterochronic parabioses, whereby the systemic circulation of two animals are joined together. Grafting muscle fragments from aged animals onto young muscle beds has been observed to result in equivalent muscle regeneration when compared with young muscle autografts (Gutmann and Carlson, 1976; Carlson and Faulkner, 1989; Roberts et al., 1997). Furthermore, Conboy et al. (2005) have demonstrated that muscle satellite cells from old mice can be rejuvenated by exposure to a young environment, by means of heterochronic parabioses. In addition, in this model satellite cells from the young animals also adopted a more aged molecular and functional state, indicating that the systemic environment is a major determinant of the “functional age” of the cell (Brack et al., 2007; Rando and Chang, 2012). Similar results have been reported by *in vitro* cell studies, where serum from young animals has been shown to rejuvenate the proliferative capacity of aged satellite cells (Carlson and Faulkner, 1989; Brack et al., 2007). In contrast, discrepant findings have been reported in human myoblasts. Whereas, some do (Carlson et al., 2009) others do not (George et al., 2010a) show that young serum can restore *in vitro* cell function of aged human myoblasts. Differences in methodology used to assess satellite cell proliferation and serum concentrations may, in part, explain these discrepant findings in human myoblasts, but future studies are warranted to further clarify these dissimilarities.

Whether or not age-related impairments in satellite cell function may have functional consequences for muscle fiber growth remains to be resolved in both animal as well as human models. Recent studies in animals have reported discrepant findings on the importance of satellite cell function during overload induced muscle fiber growth in aged mice. In a study where synergistic denervation was used to overload the *plantaris* muscle for 6 weeks in aged mice, impaired but significant muscle fiber hypertrophy was observed to be correlated to reduced satellite cell number (Ballak et al., 2015). In contrast, a study by Lee et al. (2015) reported no difference in muscle fiber hypertrophy after 2 weeks of synergistic ablation induced overload between mice in which satellite cells were depleted from skeletal muscle and wild-type controls. Although myonuclear and satellite cell content increased significantly in wild-type mice, this study reported no change in muscle fiber size during the 2 weeks of overload (Lee et al., 2015). These discrepancies between studies may be explained by the different techniques used to induce and/or duration of overload applied. More importantly, as in mice it has been reported that muscle satellite cells are only essential during extensive muscle fiber growth, it would be interesting to known whether in aged muscle satellite cell depleted mice similar results would be obtained during a more prolonged period of overload. In human skeletal muscle, the effect of age on satellite cell function is primarily investigated using acute damaging or non-damaging exercise as a form of stress to mobilize the satellite cell population. Studies have shown that expansion of the satellite cell pool is blunted during recovery from a single bout of eccentric (Dreyer et al., 2006; Walker et al., 2012) and/or resistance (McKay et al., 2012, 2013; Snijders et al., 2014c) exercise in healthy older when compared with young men (see Tables 1, 2). Moreover, we have shown that the blunted expansion of the satellite cell number during post-exercise recovery can mainly be attributed to satellite cells associated with type II muscle fibers (McKay et al., 2012; Snijders et al., 2014c). Whether this may, in part, explain the reduced capacity of older adults to increase skeletal muscle mass during prolonged resistance type exercise training remains to be fully established (Kosek et al., 2006; Petrella et al., 2006). Nevertheless, increasing our understanding on the underlying mechanisms of this impaired type II muscle fiber type specific satellite cell response during post-exercise recovery in humans is critical as this may determine whether exercise, nutritional and/or pharmaceutical interventions should specifically target these cells to combat sarcopenia.

Recent studies have suggested a number of factors that may, in part, explain the reduced satellite cell proliferative drive during post-exercise recovery observed with aging in human skeletal muscle (Figure 3). As discussed above, IL-6 has been reported to play a major role in the activation of satellite cells in response to muscle damage or hypertrophic stimuli in human skeletal muscle (Serrano et al., 2008). However, chronically elevated levels of IL-6 are associated with pro-inflammatory and muscle wasting conditions such as cachexia (Roubenoff, 1999, 2003). The infusion of IL-6 in mice leads to a catabolic environment, promote muscle wasting, and impairs muscle fiber growth (Haddad et al., 2005; Bodell et al., 2009). In humans, it is well-established that circulating IL-6 levels are systemically elevated in healthy elderly individuals (Wei et al., 1992; Ershler et al., 1993; Hager et al., 1994; McKane et al., 1994; Cohen et al., 1997; Harris et al., 1999; Visser et al., 2002; Bruunsgaard and Pedersen, 2003; Ferrucci et al., 2005; Pereira et al., 2009; McKay et al., 2013). In addition, age-related increases in serum IL-6 are associated with lower muscle strength and muscle mass in older adults (Visser et al., 2002; Pereira et al., 2009). Furthermore, in human skeletal muscle IL-6 mRNA and protein expression is significantly higher in healthy elderly compared with young individuals (McKay et al., 2013). We have previously shown a delay in the induction of IL-6 in satellite cells in response to a single bout of resistance type exercise in older adults (McKay et al., 2013). This may appear counterintuitive with the whole muscle and systemic response of IL-6, which has been reported to be more rapid and robust in older compared with young men during regeneration (McKay et al., 2013). However, basal muscle SOCS3 expression is substantially higher in old compared with young skeletal muscle (Leger et al., 2008; Alvarez-Rodriguez et al., 2012; McKay et al., 2013). The main negative regulator of IL-6/STAT3 signaling is SOCS3, which is induced by pSTAT3 as a negative feedback mechanism (Rawlings et al., 2004). Subsequently, chronically elevated IL-6 levels in older adults may impair the normal IL-6 response, and the sensitivity of satellite cells to IL-6 (McKay et al., 2013). This suggestion is further supported by elevated basal levels of SOCS3 protein and blunted increases in pSTAT3 in type II muscle fiber associated satellite cells of older adults 3 h post-exercise (McKay et al., 2013). This phenomenon is also observed in obese patients with type-2 diabetes mellitus where chronically elevated circulating IL-6 levels were associated with an impaired satellite cell response and impaired IL-6 receptor and STAT3/SOCS3 signaling cascades (Scheele et al., 2012). Therefore alterations in cytokine signaling in aging muscle may strongly influence satellite cell function.

Myostatin has been suggested to play a crucial role in the development of various muscle wasting conditions. Circulating levels of myostatin and/or expression in muscle have been reported to be elevated in animal models of cachexia (Costelli et al., 2008; Zhou et al., 2010), chronic kidney disease (Zhang et al., 2011), glucocorticoid administration (Lang et al., 2001), burn injury (Lang et al., 2001), and also during mechanical unloading (Carlson et al., 1999), and spaceflight (Lalani et al., 2000; Allen et al., 2009). Similar changes in myostatin expression have been observed in patients with cancer (Aversa et al., 2012), HIV/aids (Gonzalez-Cadavid et al., 1998), COPD (Plant et al., 2010; Hayot et al., 2011; Ju and Chen, 2012), renal failure (Sun et al., 2006), and heart failure (George et al., 2010b; Breitbart et al., 2011; Gruson et al., 2011). In healthy individuals, myostatin levels in the circulation, and/or expression in muscle have been reported to increase in response to prolonged bed rest (Reardon et al., 2001). In addition, elderly men and women have been shown to express higher levels of myostatin protein in the circulation (Yarasheski et al., 2002) and in skeletal muscle (McKay et al., 2012) compared with younger controls. In contrast, no difference exists in the proportion of myostatin^+^ satellite cells between healthy young and elderly men in the basal state (McKay et al., 2012; Snijders et al., 2014c). Yet, the decline in the number of myostatin^+^ satellite cells in response to an anabolic stimuli (e.g., single bout of resistance exercise) is significantly greater in healthy young compared with elderly men (McKay et al., 2012; Snijders et al., 2014c). The greater proportion of myostatin^+^ satellite cells during post-exercise recovery in old muscle was associated with a reduction in satellite cell activation and proliferation, as verified by the lack of progression of satellite cells into the S-phase of the cell cycle (McKay et al., 2012). Furthermore, the presence of myostatin was more prevalent in satellite cells residing in type II muscle fibers in older adults, which was associated with a delayed increase in the number of type II muscle fiber satellite cells in response to exercise (McKay et al., 2012; Snijders et al., 2014c). Hence, it was hypothesized that myostatin may inhibit satellite cell activation and proliferation to a greater degree in aged muscle, delaying the muscle adaptive response to the anabolic stimulus (McKay et al., 2012; Snijders et al., 2014c).

It would be of great interest to observe whether, for example, the irreversible dormant state of satellite cells in aged muscle observed in animals (Chakkalakal et al., 2012; Sousa-Victor et al., 2014) may explain the impaired satellite cell function observed during recovery from an anabolic stimulus and/or a period of disuse in healthy elderly people. Alleviating a pre-senescent state and/or blunted satellite cell activation response after a single exercise session in aged muscle may represent a key target in the development of future intervention strategies to more effectively counteract the progressive loss of muscle mass with aging. Future research should aim to determine the underlying mechanisms of the transition of quiescent satellite cells to their activated state in both young and elderly people in response to different anabolic stimuli.

Although it appears muscle satellite cell dysfunction is a precursor to the onset of sarcopenia, this idea remains debatable. However, it has been demonstrated that exercise training can mitigate at least some of the negative effects of aging on satellite cell number (Verdijk et al., 2009; Leenders et al., 2013) and particularly skeletal muscle as a whole (Melov et al., 2007; Lanza et al., 2008; Aagaard et al., 2010). Currently, research efforts are underway to elucidate whether exercise training actually confers a survival benefit to the satellite cells and whether exercise can restore satellite cell function either directly via modification of the proteasome or through epigenetic modifications (or both). There is an emerging body of literature that focuses on rejuvenating stem cell function through epigenetic reprogramming (recently reviewed by Rando and Chang, 2012). Although this area appears to be promising for the future, much work needs to be done to define how epigenetic states of young and old satellite cells differ and if modifications are inducible in a safe and effective manner.

## Conclusion

Over the past decade, considerable advances in our understanding of skeletal muscle satellite cells during muscle fiber repair and remodeling in human skeletal muscle have been made. Evidence from *in vivo* human studies suggests that satellite cells play a key role, not only in growth and hypertrophy but also in adaptation and remodeling in response to damaging and non-damaging exercise. Yet, it is clear that we are only at the verge of deciphering the regulatory factors underlying activation, proliferation and differentiation of satellite cells during muscle fiber repair and/or remodeling in humans. Mounting evidence indicates that aging has a profound effect on the regulation of satellite cell number and function in human skeletal muscle. As our understanding of age-related pathophysiology of satellite cells evolves the potential to treat age-related sarcopenia in a meaningful way may become a reality. It is clear, however, that examining the role of niche elements and circulating factors in addition to the satellite cell is essential in order to design therapeutic strategies to ensure potential successful and efficacious treatment of sarcopenia.

## Author contribution

TS, JN, BM, SJ, LV, LvL, GP: Manuscript writing and final approval of the manuscript.

### Conflict of interest statement

The authors declare that the research was conducted in the absence of any commercial or financial relationships that could be construed as a potential conflict of interest.

## Abbreviations

FGF2: fibroblast growth factor 2
GAS: γ-interferon activation sequence
GDF11: growth differentiation factor 11
HGF: hepatocyte growth factor
HGFA: hepatocyte growth factor activator
IGF-1: insulin-like growth factor-1
IL-6: Interleukin 6
M-Cad: M-cadherin
MGF: mechano growth factor
MRF: myogenic regulatory factor
NCAM: neural cell adhesion molecule
NSAID: non-steroidal anti-inflammatory drugs
Pax7: paired-box transcription factor 7
pSTAT3, phosphorylated: signal transducer and activator of transcription 3
TGF-β: transforming growth factor-β.
